# Toxicity assessment of hydrogen peroxide on Toll-like receptor system, apoptosis, and mitochondrial respiration in piglets and IPEC-J2 cells

**DOI:** 10.18632/oncotarget.13844

**Published:** 2016-12-09

**Authors:** Jie Yin, Miaomiao Wu, Yuying Li, Wenkai Ren, Hao Xiao, Shuai Chen, Chunyong Li, Bie Tan, Hengjia Ni, Xia Xiong, Yuzhe Zhang, Xingguo Huang, Rejun Fang, Tiejun Li, Yulong Yin

**Affiliations:** ^1^ Scientific Observing and Experimental Station of Animal Nutrition and Feed Science in South-Central, Ministry of Agriculture, Hunan Provincial Engineering Research Center of Healthy Livestock, Key Laboratory of Agro-ecological Processes in Subtropical Region, Institute of Subtropical Agriculture, Chinese Academy of Sciences, Changsha, Hunan 410125, China; ^2^ University of Chinese Academy of Sciences, Beijing 100039, China; ^3^ Department of Animal Science, Hunan Agriculture University, Changsha, Hunan 410125, China; ^4^ Hunan Co-Innovation Center of Animal Production Safety, Changsha, Hunan 410128, China; ^5^ College of Animal Science of South China Agricultural University, Guangzhou 510642, China

**Keywords:** H_2_O_2_, TLRs, apoptosis, mitochondrial respiration, piglet

## Abstract

In this study, expressions of toll-like receptors (TLRs) and apoptosis-related genes in piglets and mitochondrial respiration in intestinal porcine epithelial cells were investigated after hydrogen peroxide (H_2_O_2_) exposure. The *in vivo* results showed that H_2_O_2_ influenced intestinal expressions of TLRs and apoptosis related genes. H_2_O_2_ treatment (5% and 10%) downregulated uncoupling protein 2 (UCP2) expression in the duodenum (*P* < 0.05), while low dosage of H2O2 significantly increased UCP2 expression in the jejunum (*P* < 0.05). In IPEC-J2 cells, H_2_O_2_ inhibited cell proliferation (*P* < 0.05) and caused mitochondrial dysfunction via reducing maximal respiration, spare respiratory, non-mitochondrial respiratory, and ATP production (*P* < 0.05). However, 50 uM H_2_O_2_ significantly enhanced mitochondrial proton leak (*P* < 0.05). In conclusion, H_2_O_2_ affected intestinal TLRs system, apoptosis related genes, and mitochondrial dysfunction *in vivo* and *in vitro* models. Meanwhile, low dosage of H_2_O_2_ might exhibit a feedback regulatory mechanism against oxidative injury via increasing UCP2 expression and mitochondrial proton leak.

## INTRODUCTION

Hydrogen peroxide (H_2_O_2_), a highly reactive oxygen species (ROS), is associated with the imbalance of cellular redox *in* *vivo* and *in* *vitro* [1–4], which further induces oxidative stress and leads to irreparable oxidative injury. Various stressors have been linked to oxidative stress, such as birth process, weaning, mycotoxins contamination, and inflammatory response [5–8]. H_2_O_2_ causes a significant disruption in the oxidative balance evidenced by the decreased serum antioxidant enzymes and increased malondialdeyhde levels in various models [9–12]. However, the toxic effects of H_2_O_2_ on Toll-like receptors (TLRs), apoptosis, and mitochondrial respiration in piglet model are still obscure. Thus, in this study, piglet model and intestinal porcine epithelial cells (IPEC-J2) were used to test the toxic effects of H_2_O_2_ on TLRs system, apoptosis, and mitochondrial respiration.

## RESULTS

### Effect of H_2_O_2_ on intestinal TLRs system in piglets

In the duodenum, 10% H_2_O_2_ significantly inhibited TLR2 expression (*p* < 0.05). Compared with the control group, low dosage of H_2_O_2_ (5%) markedly upregulated TLR4 and TLR5 expression (*p* < 0.05), while 10% H_2_O_2_ inhibited TLR4 and TLR7 expression compared with 5% H_2_O_2_ (Table 1) (*p* < 0.05). In the jejunum, low dosage of H_2_O_2_ (5%) upregulated TLR1, TLR2, TLR4, TLR7, TLR10, and Myd88 expressions compared with the control group (*p* < 0.05), while the expression of TLR1, TLR3, TLR4, TLR5, TLR6, and Myd88 in the 10% H_2_O_2_ group were significantly lower than that in the low dosage of H_2_O_2_ (5%) group (*p* < 0.05) (Table 2). In the ileum, H_2_O_2_ exposure significantly inhibited TLR2 and TLR 5 expression (*p* < 0.05) (Table 3).

**Table 1:** TLRs expression in the duodenum after H_2_O_2_ exposure

Item	Cont	5% H_2_O_2_	10% H_2_O_2_
TLR1	1.00 ± 0.09	1.03 ± 0.06	0.90 ± 0.11
TLR2	1.00 ± 0.06^a^	0.88 ± 0.12^a^	0.49 ± 0.06^b^
TLR3	1.00 ± 0.11	1.28 ± 0.16	1.07 ± 0.09
TLR4	1.00 ± 0.10^b^	1.88 ± 0.16^a^	1.01 ± 0.08^b^
TLR5	1.00 ± 0.07^b^	1.54 ± 0.19^a^	1.22 ± 0.05^ab^
TLR6	1.00 ± 0.10	0.99 ± 0.07	1.00 ± 0.05
TLR7	1.00 ± 0.12^ab^	1.25 ± 0.14^a^	0.85 ± 0.07^b^
TLR8	1.00 ± 0.16	0.79 ± 0.09	0.84 ± 0.09
TLR10	1.00 ± 0.15	0.95 ± 0.09	1.14 ± 0.08
Myd88	1.00 ± 0.06	1.35 ± 0.11	1.22 ± 0.12

Data are expressed as the mean ± standard error of the mean Values in the same row with different superscripts are significant (*P* < 0.05), while values with same superscripts are not significant different (*P* > 0.05).

**Table 2:** TLR expression in the jejunum after H_2_O_2_ exposure

Item	Cont	5% H_2_O_2_	10% H_2_O_2_
TLR1	1.00 ± 0.18^b^	3.83 ± 0.41^a^	1.36 ± 0.21^b^
TLR2	1.00 ± 0.10^b^	2.39 ± 0.35^a^	1.74 ± 0.18^ab^
TLR3	1.00 ± 0.18^a^	1.00 ± 0.11^a^	0.36 ± 0.05^b^
TLR4	1.00 ± 0.08^b^	2.88 ± 0.19^a^	1.40 ± 0.13^b^
TLR5	1.00 ± 0.12^ab^	1.43 ± 0.22^a^	0.86 ± 0.15^b^
TLR6	1.00 ± 0.31^ab^	1.29 ± 0.16^a^	0.58 ± 0.09^b^
TLR7	1.00 ± 0.19^b^	1.96 ± 0.27^a^	1.21 ± 0.18^ab^
TLR8	1.00 ± 0.24	1.68 ± 0.23	1.37 ± 0.14
TLR10	1.00 ± 0.23^b^	2.51 ± 0.47^a^	1.98 ± 0.43^ab^
Myd88	1.00 ± 0.29^b^	2.58 ± 0.69^a^	0.81 ± 0.13^b^

Data are expressed as the mean ± standard error of the mean Values in the same row with different superscripts are significant (*P* < 0.05), while values with same superscripts are not significant different (*P* > 0.05).

**Table 3:** TLR expression in the ileum after H_2_O_2_ exposure

Item	Cont	5% H_2_O_2_	10% H_2_O_2_
TLR1	1.00 ± 0.14	1.00 ± 0.14	0.69 ± 0.12
TLR2	1.00 ± 0.23^a^	0.73 ± 0.06^b^	0.68 ± 0.09^b^
TLR3	1.00 ± 0.25	0.52 ± 0.06	0.53 ± 0.06
TLR4	1.00 ± 0.17	0.52 ± 0.05	1.00 ± 0.15
TLR5	1.00 ± 0.34^a^	0.42 ± 0.06^b^	0.35 ± 0.06^b^
TLR6	1.00 ± 0.25	1.17 ± 0.22	1.04 ± 0.20
TLR7	1.00 ± 0.12	1.24 ± 0.13	0.93 ± 0.17
TLR8	1.00 ± 0.24^a^	0.40 ± 0.05^b^	0.78 ± 0.16^a^
TLR10	1.00 ± 0.32	3.03 ± 0.40	2.21 ± 0.33
Myd88	1.00 ± 0.23	0.82 ± 0.17	0.90 ± 0.15

Data are expressed as the mean ± standard error of the mean. Values in the same row with different superscripts are significant (*P* < 0.05), while values with same superscripts are not significant different (*P* > 0.05).

### Effect of H_2_O_2_ on intestinal apoptosis related genes in piglets

In the duodenum, low dosage of H_2_O_2_ (5%) significantly increased Casp8 expression compared with the control group, while high dosage of H_2_O_2_ (10%) inhibited Casp8 expression compared with the low dosage of H_2_O_2_ (5%) treatment (*p* < 0.05). In the jejunum, the mRNA abundance of Fasl, Casp8, and p53 were markedly increased in the low dosage of H_2_O_2_ (5%) group compared with the control group (*p* < 0.05), while high dosage of H_2_O_2_ (10%) exposure significantly downregulated Fasl, Casp8, Bcl-2, and p53 expression (*p* < 0.05). In the ileum, H_2_O_2_ exposure inhibited Fasl, Casp3, Casp8, and Bcl-2 expression (*p* < 0.05) (Table 4).

**Table 4:** Apoptosis relative genes expression after H_2_O_2_ exposure

Item	Fasl	Casp8	Casp3	Bcl2	P53
Duodenum					
Cont	1.00 ± 0.13	1.00 ± 0.10^b^	1.00 ± 0.08	1.00 ± 0.09	1.00 ± 0.07
5% H_2_O_2_	1.05 ± 0.17	1.96 ± 0.27^a^	1.00 ± 0.09	0.91 ± 0.11	1.28 ± 0.17
10% H_2_O_2_	0.90 ± 0.10	1.32 ± 0.12^b^	0.91 ± 0.07	1.04 ± 0.11	1.28 ± 0.12
Jejunum					
Cont	1.00 ± 0.18^b^	1.00 ± 0.25^b^	1.00 ± 0.16	1.00 ± 0.17^ab^	1.00 ± 0.16^b^
5% H_2_O_2_	1.71 ± 0.27^a^	2.11 ± 0.30^a^	1.58 ± 0.41	1.47 ± 0.22^a^	2.42 ± 0.36^a^
10% H_2_O_2_	1.12 ± 0.16^b^	0.84 ± 0.14^b^	0.83 ± 0.16	0.78 ± 0.10^b^	0.98 ± 0.14^b^
Ileam					
Cont	1.00 ± 0.26^a^	1.00 ± 0.18^a^	1.00 ± 0.17^a^	1.00 ± 0.19^a^	1.00 ± 0.09^ab^
5% H_2_O_2_	0.37 ± 0.07^b^	0.50 ± 0.06^b^	0.16 ± 0.02^b^	0.55 ± 0.07^b^	0.82 ± 0.07^b^
10% H_2_O_2_	0.25 ± 0.07^b^	0.73 ± 0.10^ab^	0.33 ± 0.05^b^	0.60 ± 0.09^b^	1.24 ± 0.12^a^

Data are expressed as the mean ± standard error of the mean. Values in the same row with different superscripts are significant (*P* < 0.05), while values with same superscripts are not significant different (*P* > 0.05).

### Effect of H_2_O_2_ on intestinal UCP2 expression in piglets

**Figure 1:**
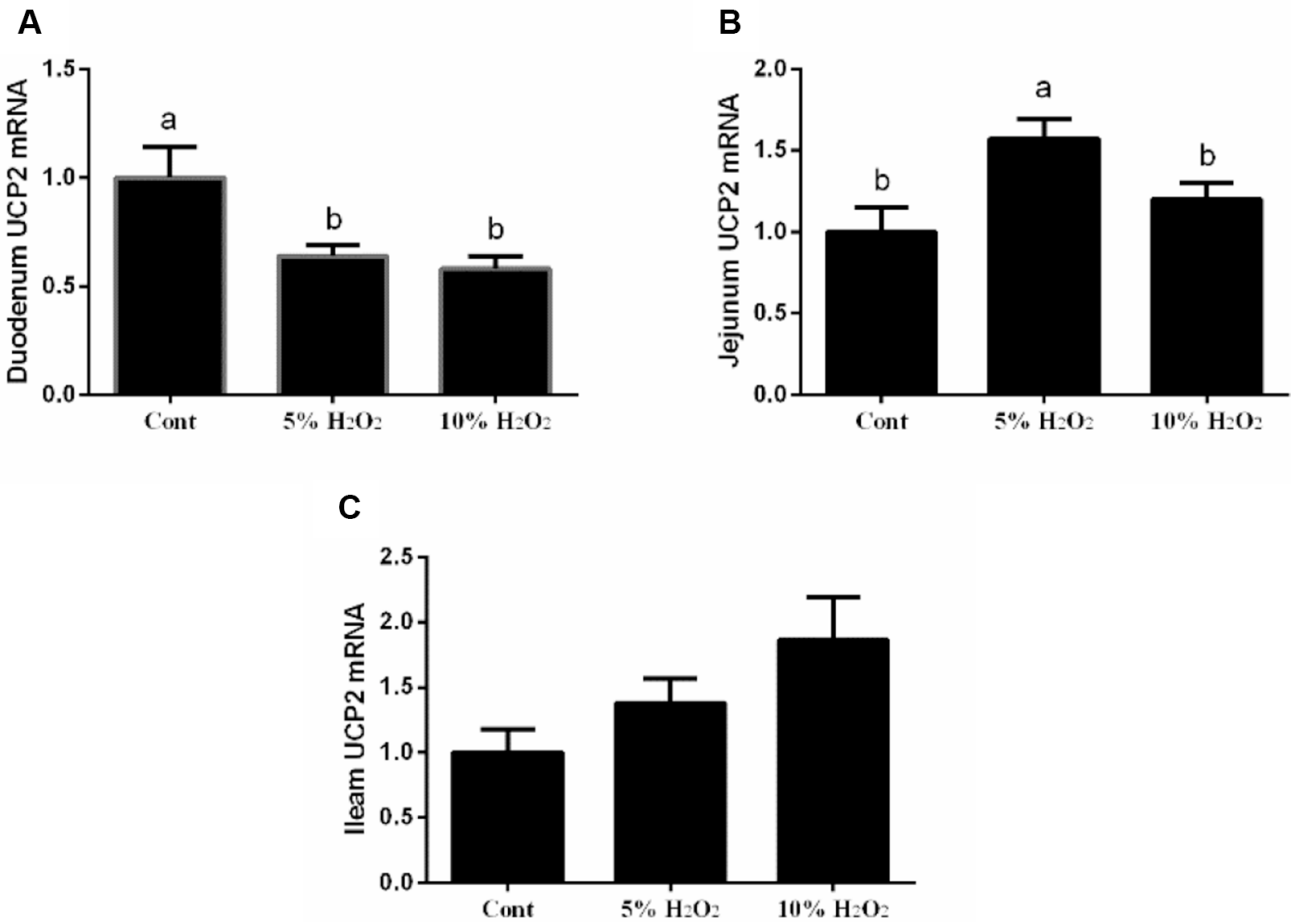
Effects of H_2_O_2_ on UCP2 expression in the duodenum (**A**), jejunum (**B**), and ileum (**C**) (*n* = 6). Data are expressed as the mean ± standard error of the mean. Values in the same row with different superscripts are significant (*P* < 0.05), while values with same superscripts are not significant different (*P* > 0.05).

In the duodenum, 5% and 10% H_2_O_2_ administration markedly decreased UCP2 expression compared with the control group. In the jejunum, low dosage of H_2_O_2_ (5%) treatment upregulated UCP2 expression, while high dosage of H_2_O_2_ (10%) treatment reduced UCP2 upregulation compared with low dosage of H_2_O_2_ (5%) treatment (*p* < 0.05) (Figure 1).

### Effect of H_2_O_2_ on cell proliferation in IPEC-J2 cells

**Figure 2:**
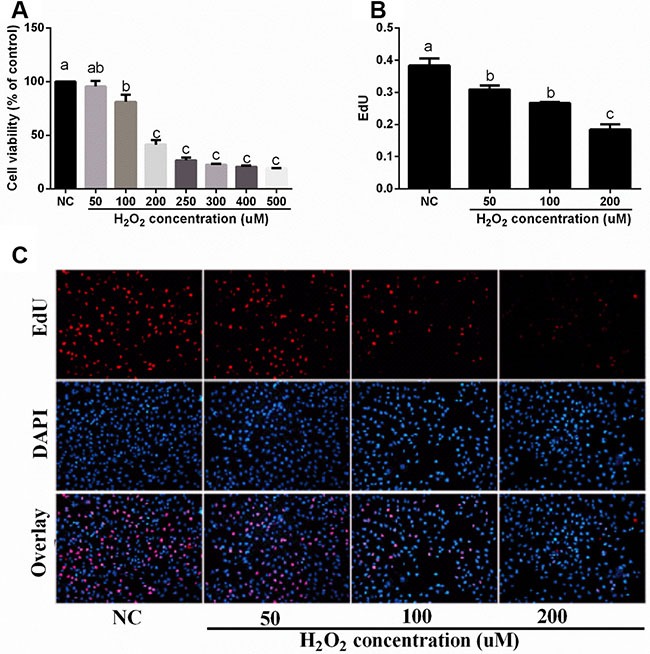
Effects of H2O2 on cell viability and proliferation in IPEC-J2 cells (n = 4 or 6) (**A**) cell viability (%); (**B**) EdU content; and (**C**) EdU results (×20). Data are expressed as the mean ± standard error of the mean. Values in the same row with different superscripts are significant (*P* < 0.05), while values with same superscripts are not significant different (*P* > 0.05).

As shown in Figure 2A, H2O_2_ (100, 200, 250, 300, 400, and 500 uM) significantly inhibited cell viability. The results from EdU assay also showed that H_2_O_2_ (50, 100, and 200 uM) exposure significantly reduced cell proliferation (Figure 2B and 2C).

### Effect of H_2_O_2_ on cell mitochondrial respiration in IPEC-J2 cells

**Figure 3:**
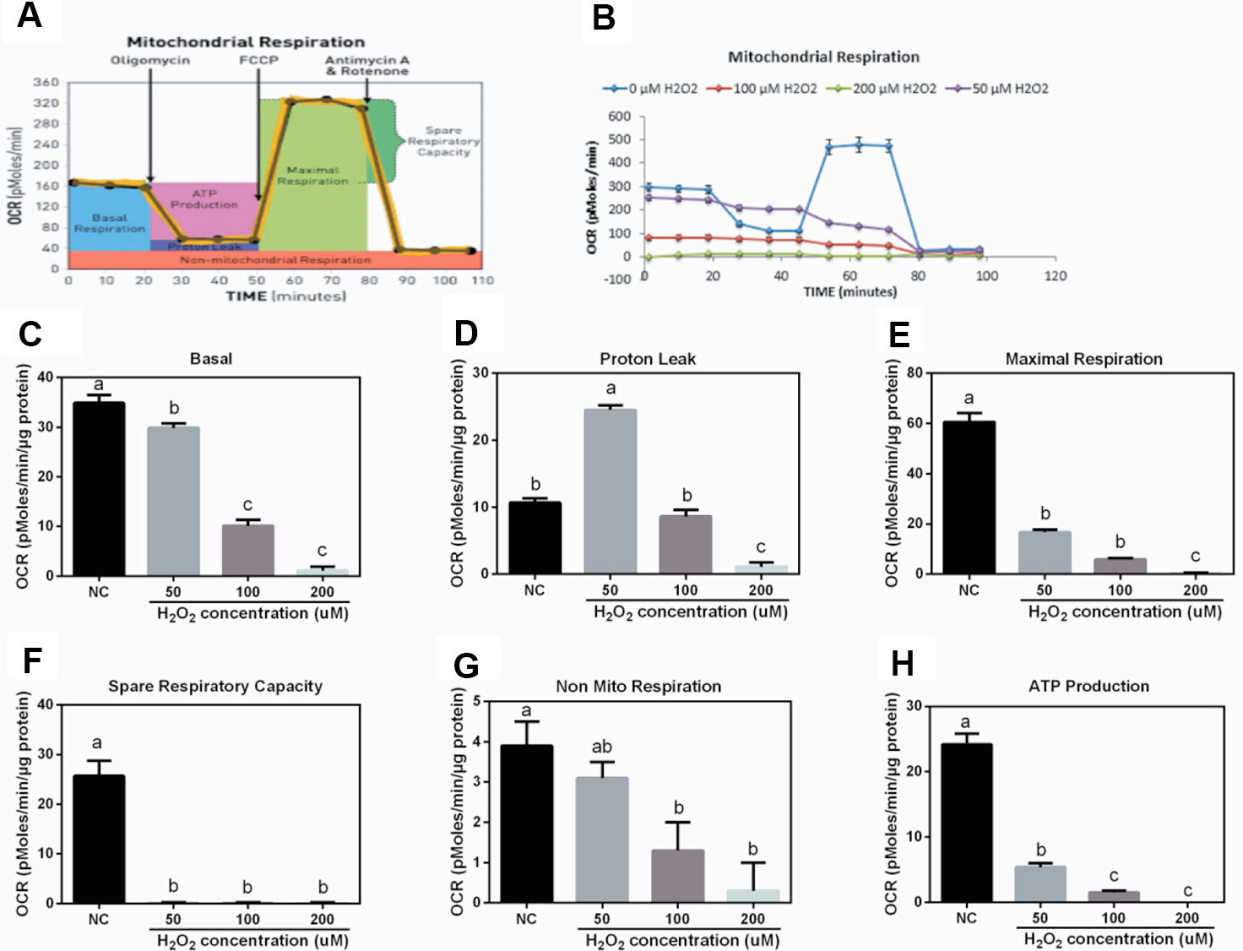
Effects of H2O2 on mitochondrial respiration in IPEC-J2 cells (n = 4) (**A**) Assay working model; (**B**) mitochondrial respiration curve; (**C**) basal respiration; (**D**) proton leak; (**E**) maximal respiration; (**F**) spare respiratory; (**G**) non-mito respiratory; and (**H**) ATP production. Data are expressed as the mean ± standard error of the mean. Values in the same row with different superscripts are significant (*P* < 0.05), while values with same superscripts are not significant different (*P* > 0.05).

The working model of mitochondrial respiration determination was shown at Figure 3A and 3B. The results showed that H_2_O_2_ decreased mitochondrial basal OCAR (Figure 3C), maximal respiration (Figure 3E), spare respiratory (Figure 3F), non-mitochondrial respiratory (Figure 3G), and ATP production (Figure 3H) in a dosage-dependent manner. Interestingly, 50 uM H_2_O_2_ significantly increased mitochondrial proton leak compared with the control group (Figure 3D), while high dosage of H_2_O_2_ (200 uM) markedly inhibited mitochondrial proton leak compared with other dosage groups.

## DISCUSSION

Previous studies revealed that intragastric or peritoneal injection of H_2_O_2_ induced inestinal oxidative stress. Meanwhile, the dysfunction of intestinal permeability, morphology, and barrier function were noticed after exposure to H_2_O_2_ in piglets and mice [9, 13–15]. In this study, we further found that H_2_O_2_ affected inestinal expression of TLR system and apoptosis related genes in piglets and influenced mitochondrial respiration in IPEC-J2 cells.

TLRs (TLR 1–10) are expressed by various cells in the gastrointestinal tract and involve in the induction of an inflammatory response and oxidative stress [16–19]. Previous studies exhibited that H_2_O_2_ exposure induced intestinal oxidative stress and inflammation [9, 13, 20, 21]. In this study, we found that low dosage of H_2_O_2_ (5%) upregulated TLRs, including TLR4 and TLR5 in the duodenum and TLR1, TLR2, TLR3, TLR4, TLR7, TLR10, and Myd88 in the jejunum. However, high dosage of H_2_O_2_ (10%) inhibited TLR2, TLR4, and TLR7 in the jejunum and TLR1, TLR3, TLR4, TLR5, TLR6, and Myd88 in the jejunum compared with the low dosage of H_2_O_2_ treatment. Thus, we speculated that low H_2_O_2_ might activate TLRs while high H_2_O_2_ inhibited TLRs. Meanwhile, the effect may be segmental dependent because H_2_O_2_ downregulated TLR2 and TLR5 expression in the ileum.

Our previous study showed that H_2_O_2_ exposure caused intestinal morphologic injury [9], which may associate with apoptosis. In the present study, we found that H_2_O_2_ treatment influenced intestinal Fasl, Casp3, Casp8, Bcl-2, and p53 expressions in piglets. This hypothesis is further confirmed by the CKK-8 and EdU assay that H_2_O_2_ exposure markedly inhibited cell proliferation. Apoptosis and proliferation play a crucial role in cell growth and oxidative stress [22–24].

UCP2 has been considered as a feedback regulatory mechanism for oxidative stress and our previous studies showed that birth and weaning-induced oxidative stress activated UCP2 to improve antioxidant function [5, 6]. In this study, H_2_O_2_ exposure inhibited UCP2 expression in the duodenum, while low dosage of H_2_O_2_ upregulated UCP2. In the jejunum, low dosage of H_2_O_2_ enhanced UCP2 mRNA abundance, while high dosage markedly inhibited UCP2 expression. So we speculated that low dosage of H_2_O_2_ might exhibit a feedback regulatory mechanism against oxidative stress evidenced by upregulating UCP2 expression. Furthermore, consistent with this speculation, *in vitro* data suggested that low dosage of H_2_O_2_ (50 uM) markedly enhanced mitochondrial proton leak. UCP2 has been reported to increase proton leak, which further decreases ROS production and protects against oxidative stress [25]. The mitochondrial respiration assay further confirmed the feedback regulatory mechanism of low dosage of H_2_O_2_ against oxidative stress via increasing UCP2 expression and mitochondrial proton leak.

Mitochondrion not only plays a crucial role in the generation, sensing, and scavenging of ROS [26], but also tightly linked to apoptosis and proliferation. The present results showed that H_2_O_2_ reduced mitochondrial basal OCR, maximal respiration, spare respiratory, non-mito respiratory, and ATP production in a dosage-dependent manner in a dosage-dependent manner. Similarly, Rose et al. reported that oxidative stress induced mitochondrial dysfunction via affecting ATP-linked respiration and maximal respiratory capacity [27].

In conclusion, H_2_O_2_ affected intestinal TLRs system and apoptosis related genes, the effect exhibited dosage and tissue dependent. Meanwhile, H_2_O_2_ induced mitochondrial dysfunction. However, low dosage of H_2_O_2_ stimulation might exhibited a feedback regulatory mechanism against oxidative injury via increasing UCP2 expression and mitochondrial proton leak.

## MATERIALS AND METHODS

### Animal surgery and experimental design

Animal surgery was conducted according to our previous report [9]. Briefly, eighteen healthy piglets of similar bodyweight (Landrace× Large White) (ZhengHong Co., China) were anesthetized (Zoletil 50, Virbac Co., France) and then operated to install a silicone coated latex T-shape catheter (Zhan Jiang Star Enterprism Co., China) in the helicobacter. After surgery, all piglets recovered uneventfully for a week, then randomly divided into three groups (*n* = 6): a control group in which piglets received an intragastric administration via the T-shape catheter of 10 mL/10 kg PBS buffer; a 5% H_2_O_2_ group in which piglets were given an intragastric administration of 5% H_2_O_2_; a 10% H_2_O_2_ group in which piglets received an intragastric administration of 10% H_2_O_2_ [9]. All piglets were allowed free access to water and feed throughout the experimental period.

All piglets were killed after 7 days. 3 cm middle duodenum, jejunum, and ileum samples were harvested and immediately frozen in liquid nitrogen for subsequent analyses. This study was approved by the animal welfare committee of the Institute of Subtropical Agriculture, Chinese Academy of Sciences.

### cDNA synthesis and quantification mRNA by real-time PCR analysis

Extraction of total RNA and its reverse transcription were performed according to our previous reports [10, 11]. Primers were designed with Primer 5.0 according to the gene sequence of pig (http://www.ncbi.nlm.nih.gov/pubmed/) to produce an amplification product (Table 5). β-actin was used as a housekeeping gene to normalize target gene transcript levels. Real-time PCR was performed according to our previous study [10]. Relative expression was normalized and expressed as a ratio to the expression in control group.

**Table 5:** Primers used in this study

Genes	Nucleotide sequence of primers (5′–3′)
β-Actin	F: CTGCGGCATCCACGAAACT
	R: AGGGCCGTGATCTCCTTCTG
UCP2	F: CCAATGTCGCTCGTAATG
	R: TGGCAGGGAAGGTCATC
p53	F: CTGCTTCCTGAAAACAACC
	R: AAGGGACAAAGGACGACA
Casp-3	F: AGCATCCACATCTGTACCA
	R: CCGGAATGGCATGTCGAT
Casp-8	F: TGGGAAAGCATATGAGCTATTCAA
	R: TTCCGGAGTCATCTGTGAGTGA
Bcl-2	F: ACGGTGGTGGAGAGACTCTTCA
	R: TGACGCTCTCCACACACATGAC
FasL	F: AATGGGAAGACACCTATGGAA
	R: CTTAGAGCTTATATAAGCCGAAAAACGTC
TLR1	F: TGCTGGATGCTAACGGATGTC
	R: AAGTGGTTTCAATGTTGTTCAAAGTC
TLR2	F: TCACTTGTCTAACTTATCATCCTCTTG
	R: TCAGCGAAGGTGTCATTATTGC
TLR3	F: AGTAAATGAATCACCCTGCCTAGCA
	R: GCCGTTGACAAAACACATAAGGACT
TLR4	F: GCCATCGCTGCTAACATCATC
	R: CTCATACTCAAAGATACACCATCGG
TLR5	F: CAGCGACCAAAACAGATTGA
	R: TGCTCACCAGACAGACAACC
TLR6	F: AACCTACTGTCATAAGCCTTCATTC
	R: GTCTACCACAAATTCACTTTCTTCAG
TLR7	F: TCAGTCAACCGCAAGTTCTG
	R: GATGGATCTGTAGGGGAGCA
TLR8	F: AAGACCACCACCAACTTAGCC
	R: GACCCTCAGATTCTCATCCATCC
TLR10	F: CACGACAGCCGAATAGCAC
	R: GGGAACAGGGAGCAGAGC
Myd88	F: CCTGTCCAACTGCCTCATTTG
	R: CTAAGTGTTCTAAGGATGTGTTTCTG

F: forward; R: reverse.

### Cell culture

Intestinal porcine epithelial cells (IPEC-J2) were cultured in serial passage in uncoated plastic culture flasks (100 mm^2^) in DMEM-H containing 10% FBS, 5 mM l-glutamine, 100 U/ml penicillin, and 100 μg/ml streptomycin. Cells were treated with different dosage of H_2_O_2_ to induce oxidative stress. Cell viability was evaluated with the CKK-8 assay (Sigma–Aldrich) according to the manufacturer's instructions. Briefly, 8 × 10^3^ cells were seeded in 96-well plates. The following day, cells were incubated with 50, 100, 200, 250, 300, 400, and 500 uM H_2_O_2_ for 4 hours and then assayed.

### EdU (5-Ethynyl -2′- deoxyuridine) measurement

IPEC-J2 cells cultured in 96-well plates after 96 hour incubation were labeled with 50 μM 5-ethynyl-2′-deoxyuridine (EdU; Invitrogen) for 1 hour (pulse) before replacing with fresh medium. Cell fixation, permeabilization and EdU detection were performed following manufacturer's instructions for EdU kit (Invitrogen). Cells were measured using an inverted fluorescence microscope (DMI3000B, Leica, Germany).

### Mitochondrial respiration

Mitochondrial respiration after H_2_O_2_ exposure was measured via the XF-24 Extracellular Flux Analyzer and Cell Mito Stress Test Kit. Oligomycin, arbonyl cyanide-p-trifluoromethoxyphenylhydrazone (FCCP), rotenone and antimycin A were used to estimate the contribution of non-ATP–linked oxygen consumption (proton leak), ATP–linked mitochondrial oxygen consumption (ATP production), and maximal respiration capacity. The spare respiratory capacity was represented by the maximal respiratory capacity subtracted from the baseline oxygen consumption rate (OCR). The residual oxygen consumption that occurred after addition of rotenone and antimycin A was ascribed to non-mitochondrial respiration and was subtracted from all measured values in the analysis [12]. Total cellular protein was determined and used to normalize mitochondrial respiration rates.

### Statistical analysis

All statistical analyses were performed by using the one-way analysis of variance (ANOVA) to test homogeneity of variances via Levene's test and followed with Tukey's multiple comparison test (SPSS 17.0 software). Data are expressed as the mean ± standard error of the mean. Values in the same row with different superscripts are significant (*P* < 0.05), while values with same superscripts are not significant different (*P* > 0.05).
